# Supplementary material for: DMRforPairs: identifying Differentially Methylated Regions between unique samples using array based methylation profiles
Source: BMC Bioinformatics. 2014 May 15;15:141. doi: 10.1186/1471-2105-15-141 (PMC4046028; doi:10.1186/1471-2105-15-141)
Supplement: Additional file 2 — DMRforPairs output for the comparison of A431-MCF7 and NA17018-NA17105. Please start from the HTML files in each folder. Available via the BMC Bioinformatics website. [file 1471-2105-15-141-S2.zip › 1394847754114233_MOESM2_ESM/A431_MCF7/figures/11849.pdf]

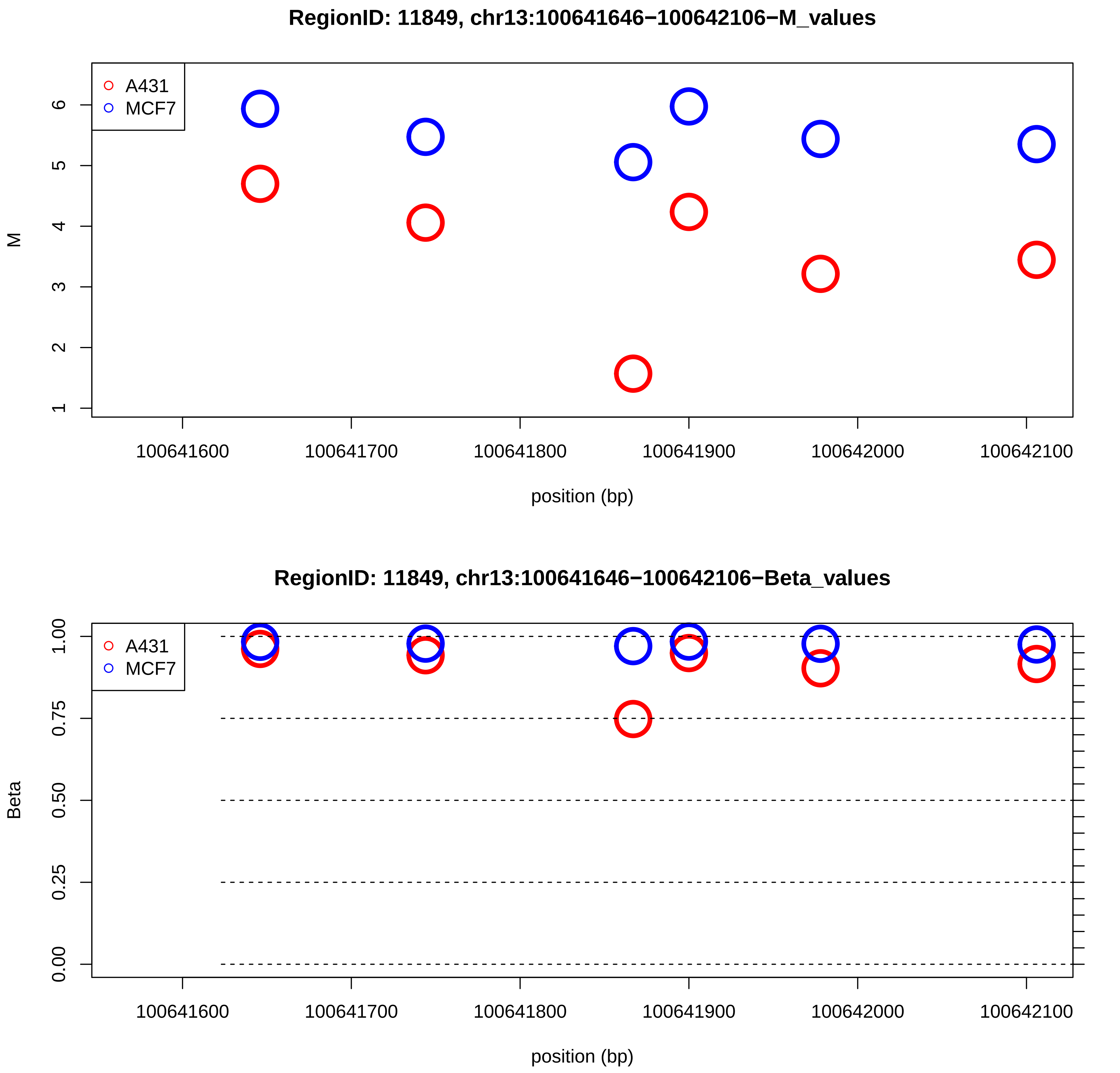

RegionID: 11849, chr13:100641646−100642106−M_values
A431
MCF7
M
1
2
3
4
5
6
100641600
100641700
100641800
100641900
100642000
100642100
position (bp)
RegionID: 11849, chr13:100641646−100642106−Beta_values
A431
MCF7
Beta
0.00
0.25
0.50
0.75
1.00
100641600
100641700
100641800
100641900
100642000
100642100
position (bp)

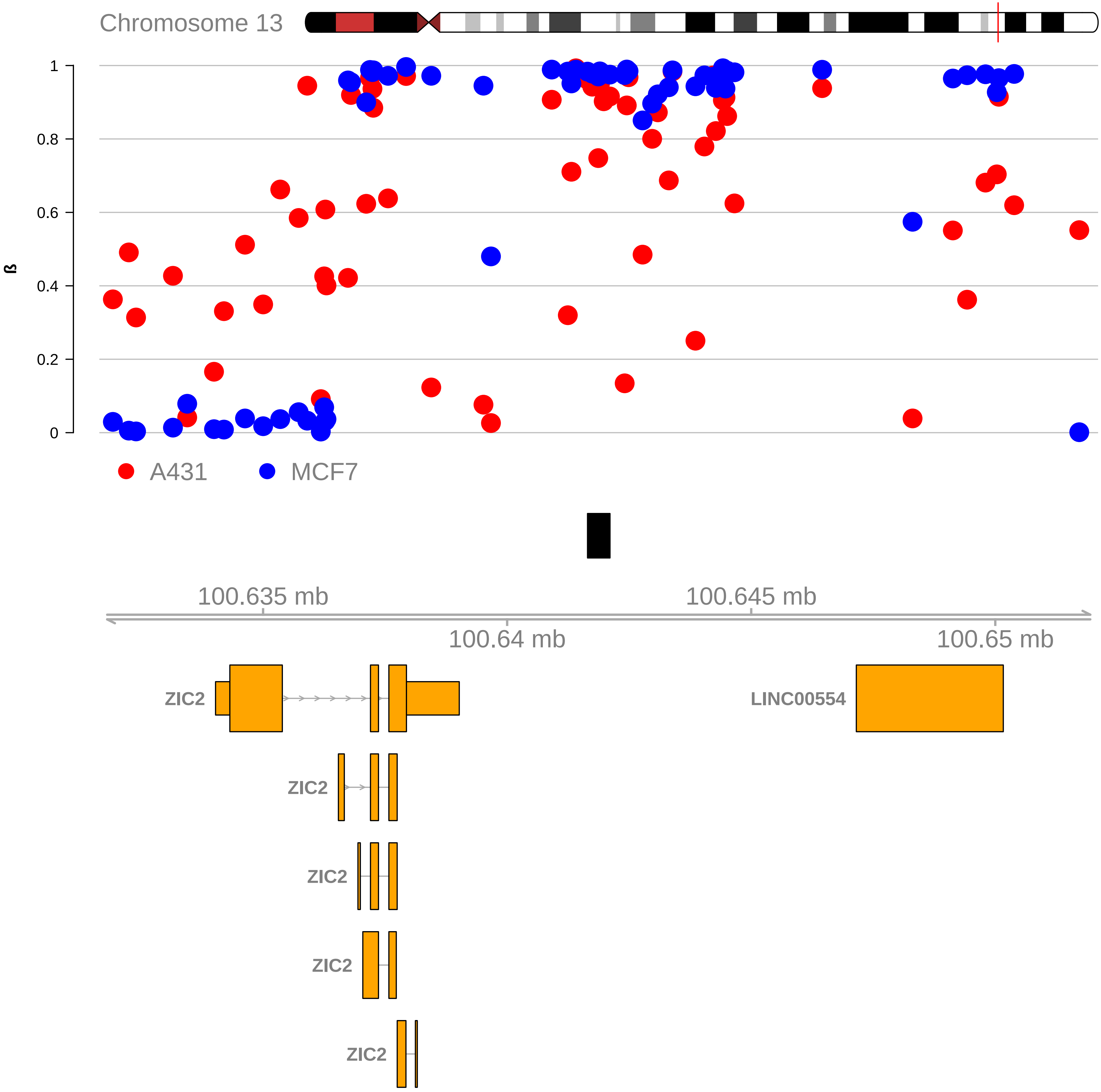

Chromosome 13
1
0.8
0.6
0.4
0.2
0
ß
A431
MCF7
100.635 mb
100.64 mb
100.645 mb
100.65 mb
ZIC2
LINC00554
ZIC2
ZIC2
ZIC2
ZIC2
